# A comprehensive review of artificial intelligence as a catalyst in aging research: insights, gaps and future perspectives

**DOI:** 10.3389/fragi.2026.1644669

**Published:** 2026-01-28

**Authors:** Tasnuva Binte Mahbub, Parsa Safaeian, Salman Sohrabi

**Affiliations:** Department of Bioengineering, University of Texas at Arlington, Arlington, TX, United States

**Keywords:** aging, artificial intelligence, clinical translatability, in-vivo validation, model organisms

## Abstract

Aging is driven by interconnected genetic, epigenetic, molecular, and physiological processes spanning from unicellular to organismal levels. The surge in high-throughput data, from clinical and imaging to multi-omics, has outpaced traditional analysis methods; driving the integration of artificial intelligence (AI) into aging research. This comprehensive review examines the application of machine learning, deep learning, and computer vision across four canonical aging models (yeast, *Caenorhabditis elegans*, *Drosophila melanogaster*, and mice), highlighting AI’s role in lifespan prediction, biomarker and gene discovery, aging-clock construction, and assay automation via automated animal counting and imaging. However, only 3% of the reviewed studies incorporated *in vivo* biological validation with common issues including small and imbalanced datasets, dataset bias, prediction noise, lack of cross-species analyses, absence of cytotoxicity testing, and overreliance on synthetic data. These drawbacks pose AI as just an aiding tool rather than a standalone solution, and without improvements in these sectors, AI-derived findings should be considered hypothesis generating rather than definitive conclusions. To address these issues, we propose the development of a standardized scoring system, AI Quality Assessment Metric (AI-QAM), for aging research that will evaluate studies on six criteria: (1) dataset size, (2) feature dimensionality, (3) biological validation type, (4) species diversity, (5) model generalizability, and (6) interpretability. Moreover, to mitigate the problem of lacking a unifying of a framework integrating AI approaches with biological mechanisms of aging, we present a conceptual framework, mapping AI applications across biological levels and aging hallmarks. AI will fulfill its potential in aging research only when it is firmly grounded in biological principles, systematically benchmarked, and rigorously validated through experimental studies.

## 1 Introduction

Aging is a complex biological process involving interconnected molecular, cellular, physiological, and tissue-level changes that over time contribute to functional decline (Chen J.-C. et al., 2024; Peysselon and Ricard-Blum, 2011). As the global elderly population continues to grow, there is an urgent need to understand the mechanisms of aging and develop strategies to promote healthy aging and prevent age-related diseases (Aman et al., 2020; Bernal et al., 2024; Lin Y. et al., 2023). Model organisms such as *Caenorhabditis elegans*, *Drosophila melanogaster*, yeast, and mice have become central to aging studies due to their short lifespans, genetic tractability, and biological relevance to human systems. These models offer experimental scalability and ethical advantages, making them powerful tools for investigating conserved aging mechanisms.

Research in these organisms has traditionally relied on labor-intensive, time-consuming assays to study aging phenotypes. However, the growing use of high-throughput techniques, such as next-generation sequencing, omics profiling, and advanced imaging, have dramatically increased the volume and complexity of data generated from both model organisms and human clinical studies (Bernal et al., 2024; Lin Y. et al., 2023; Mishra and Li, 2020). Managing and interpreting this data manually is highly inefficient, prompting a shift toward computational tools capable of processing and interpreting these data.

Artificial intelligence (AI) has rapidly emerged as a transformative approach in this context. Techniques such as machine learning (ML), deep learning (DL), and computer vision (CV) are now applied to identify aging-related pathways, predict therapeutic targets, construct aging clocks, and enhance experimental (Marino et al., 2023; Zhavoronkov et al., 2019; Zhavoronkov and Mamoshina, 2019). AI not only accelerates discovery but also enables automation of previously manual tasks, reducing error and increasing reproducibility.

Despite its growing impact, AI remains a tool, powerful but not without limitations. Studies frequently face issues such as small or biased datasets, overfitting, lack of experimental validation, and poor generalizability across species. Addressing this gap, we proposed a unifying framework where AI applications (prediction, discovery, automation, etc.) are mapped across biological organization levels (molecular, cellular, tissue, and organismal) and mentionable aging hallmarks (e.g., genomic instability, epigenetic alterations, mitochondrial dysfunction). This map clarifies how specific AI tools, such as CNNs for image-based phenotype prediction or ensemble ML for transcriptomic aging clocks, are better suited for different mechanistic aspects of aging. Moreover, we introduced a 3-tiered validation rubric to claim the AI-derived findings as translational and encouraged the development of standardized AI-QAM (AI Quality Assessment Metric), a scoring system to evaluate research robustness. Thus, this review explored how AI has been integrated into aging research across five model systems-yeast, *C. elegans*, *Drosophila*, mice, and humans, highlighted both its transformative contributions and the persistent challenges regarding reliable translation, and provided structure and direction for future work in AI-driven gerontology.

## 2 Search strategy

A structured literature search was conducted to review all the aging-related studies available AI across five biological systems: yeast, *C. elegans*, *Drosophila*, mice, and humans. The studies were collected from major academic databases, including PubMed and Google Scholar, by searching with the keywords like AI in aging studies, aging related keywords (like lifespan, healthspan, reproductive span) and AI methods related keywords (like machine learning, deep learning, computer vision) along with mentioning model organisms. The studies using AI for any aging-related research and prominent result generation have been included in this literature after a thorough review. The extracted information from each included study was the organism, data type used to train the model (e.g., imaging, omics, clinical), modeling approach, validation strategy and the outcome of the research. A total of 55 studies (yeast: 8, *C. elegans*: 15, *Drosophila*: 9, mice: 11, and humans: 12) were reviewed. Biological validation was quantified in a three-level hierarchy: computational (*in silico* only), *in vitro*, and *in vivo*, based on what each study explicitly reported.

## 3 Overview of artificial intelligence

**FIGURE 1:**
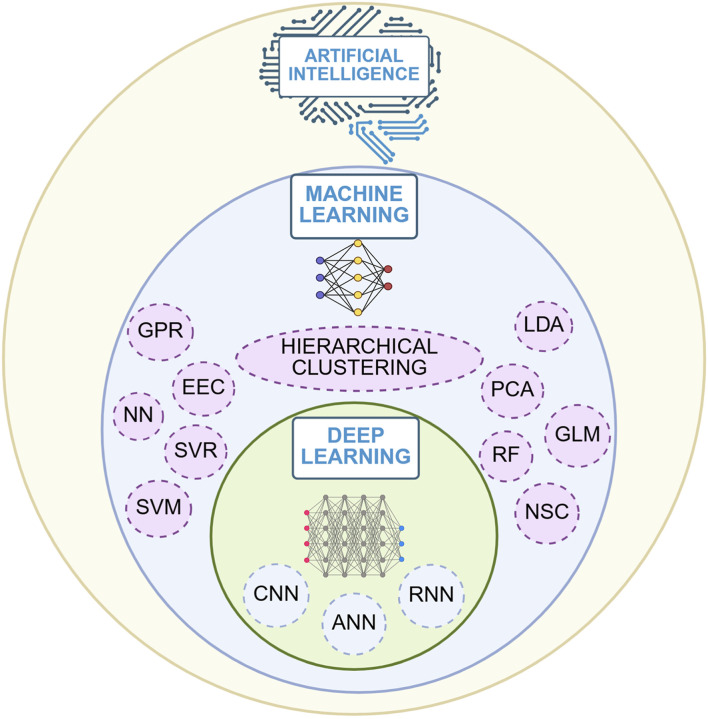
Different Domains of AI (Created in https://BioRender.com). Abbreviations: NN, Neural Network; ANN, Artificial Neural Network (a subset of neural network); CNN, Convolutional Neural Network; RNN, Recurrent Neural Network; RF, Random Forest; SVM, Support Vector Machine; GPR, Gaussian Process Regression; EEC, Evolutionary Ensemble Classifier; SVR, Support Vector Regression; LDA, Linear Discriminant Analysis; PCA, Principal Component Analysis; GLM, Generalized Linear Model; NSC, Nearest Shrunken Centroid.

In the quest for simpler analysis, interpretation, prediction, and decision-making from the vast volumes of data generated in today’s digital world, researchers have increasingly turned to AI. AI is a multidisciplinary field that develops systems capable of simulating human cognitive functions, such as perception, prediction, decision-making, and autonomous actions. It extends beyond the capabilities of traditional statistical approaches in terms of data analysis and data interpretation (Mishra and Li, 2020; Moore et al., 2019), which stands as a strong basis for researchers to opt for AI in their research. AI systems leverage sophisticated computational techniques, including ML, DL and CV. As hierarchically illustrated in Figure 1, CV is a subset of DL, which itself falls under ML. ML detects complex patterns in large, nonlinear, multimodal datasets, whereas DL uses multi-layered neural networks for complex feature extraction. CV is a DL-driven model for visual data interpretation and analysis (Bernal et al., 2024).

## 4 AI in aging research: how AI transforms biomedical discovery

AI in aging research gained momentum since 2020 and continues to play a transformative role in the field (Leung et al., 2024). The most prominent advantage that AI offers is the potentiality of pattern recognition with no requirement of preceding biological knowledge, but the quality and structure of input data remain crucial (Zhavoronkov et al., 2019). With advancements in technology, vast amounts of clinical data (including patient records, imaging data, and multi-omics datasets) are now well-preserved and accessible for research. Analyzing these data manually to uncover trends or develop novel therapeutics is not only time-consuming but also prone to human error. AI streamlines this process, integrating massive datasets to generate predictive models and identify patterns efficiently. For instance, traditional aging research often involves extensive wet-lab experiments to test individual compounds for their potential anti-aging effects. AI significantly simplifies this process by predicting optimal target compounds, thereby reducing the need for exhaustive trial-and-error experiments (Fabris et al., 2017). This accelerates research, reduces costs, and minimizes labor-intensive efforts.

While AI makes invaluable contributions to aging research, can it be fully trusted? The performance of AI heavily relies on input data quality, dataset size and biasness. Moreover, synthetic data generation, lack of biological experimental validation and generalization of model across species are some of the drawbacks that raise questions about the reliability of AI models for drawing major biological conclusions. In this review, we highlight AI’s role as a powerful tool in aging research, survey recent AI-driven advancements, and discuss the benefits and challenges of its integration.

## 5 Model organisms in aging study

Aging is a universal and inherent characteristic of virtually all living organisms, tissues, and cells (Zhavoronkov et al., 2019). Given the complexity of human biology, the long lifespan, and ethical constraints associated with aging research in humans, model systems are widely employed as analogs. These systems enable *in vivo* experiments that offer precise insights into the molecular and cellular mechanisms underlying aging (Lin et al., 2023). The genetic mechanisms regulating lifespan in these models have profound implications for understanding human longevity. Research has uncovered key signaling pathways and gene networks linked to aging, which is summarized in Table 1. Thus, studying aging in these model systems has proven to be precise, interpretable, and cost- and time-effective, offering invaluable insights into the biology of aging across species.

**TABLE 1:** Aging-related genes and pathway conserved across model organisms.

Model organism	Key genes/pathways	Human orthologs	References
*C.elegans*	daf-2 (insulin/IGF-1 receptor homolog)	IGF1R	Kenyon (2011)
	daf-16 (FOXO transcription factor)	FOXO Family	
Yeast	TOR pathway	mTOR	Kaeberlein et al. (2004), Powers et al. (2006)
	SIR2	SIRT1–SIRT7 family	
*Drosophila melanogaster*	dSir2	Sirtuin 2	Rogina and Helfand (2004)

**FIGURE 2:**
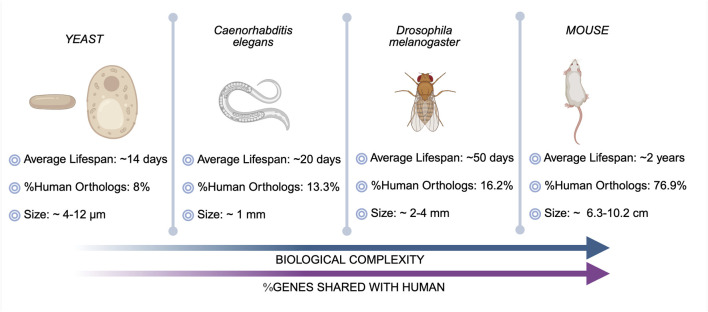
Key model organisms in aging research (created in https://BioRender.com).

Among the various model organisms used in research, the most extensively studied include yeast, *C. elegans*, *Drosophila*, and mice. In terms of biological complexity (nervous system, brain, interactive system between cells and tissues, and structural complexity) and human orthologs (protein-coding genes), these models can be arranged in a hierarchy, as illustrated in Figure 2. The hierarchy begins with the unicellular yeast with approximately 8% orthologs, followed by the multicellular *C. elegans* with 13.3% orthologs and a simple nervous system with a well-defined genetic structure. Next in complexity is *Drosophila* sharing of 16.2% of human genes, which has a more advanced nervous system and a higher degree of genetic regulation. At the highest level of complexity are mice comprising of around 76.9% orthologs, which share similar organ systems and brain structures with humans, making them highly relevant for biomedical research (Marzi et al., 2023; Osborne et al., 2020). While simpler model organisms like yeast and *C. elegans* offer advantages such as ease of genetic manipulation, lower cost, and rapid experimental outcomes, more complex models like *Drosophila* and mice provide greater physiological relevance and translational potential for human diseases, enabling more reliable validation of therapeutic strategies.

### 5.1 Yeast

Yeast, particularly *Saccharomyces cerevisiae* (Baker’s/Budding Yeast) and *Schizosaccharomyces pombe* (Fission Yeast), are premier unicellular models for dissecting aging at the molecular level (Qin, 2019). Their simplicity, miniscule size (∼4–12 µm), rapid lifespan (∼14 days) (Osborne et al., 2020), genetic modifiability, fully annotated genomes combined with ∼86 yeast genes among 6,000 directly modulating longevity (Chen et al., 2013), and significant genetic similarities to humans make them ideal for high-resolution studies. Notably, *S. cerevisiae* has been instrumental in identifying key aging-related pathways, including sirtuins and the TOR signaling pathway, which are conserved across species (Ghafari et al., 2022). The key assays in yeast aging research include replicative lifespan (RLS), measuring the total number of cell divisions a mother cell undergoes until senescence (Ghafari et al., 2021), and chronological lifespan (CLS), assessing how long a yeast cell survives in a non-dividing state after depleting available nutrients (Mirisola and Longo, 2022). Some major contributions of AI in aging-related studies using yeast as a model organism are discussed below.

#### 5.1.1 AI-driven target identification and gene discovery

AI has been widely applied to uncover not only longevity-related genes but also to analyze protein interactions and gene functions (Sinclair, 2002). In this respect, (Huang et al., 2012), developed a two-layer predictive algorithm with a nearest neighbor (NN) ML model, to assess the impact of gene deletions on *Saccharomyces cerevisiae* lifespan. By analyzing genomic datasets, they underscored the critical roles of mitochondrial function and chromatin silencing in lifespan regulation (Chen et al., 2013). focused on discovering previously unidentified longevity-associated genes employing hierarchical clustering to categorize genes based on their microarray expression profiles. This method successfully identified six highly probable longevity genes, along with one already recognized longevity gene, IDH1, validating the robustness of their algorithm in prediction. More recently, (Rodríguez-López et al., 2023), combined large-scale colony-growth phenomics with the NET-FF ML (Neural Network for Time-Series Forecasting and Feature Fusion) framework in *S. pombe* with the aim of functional protein discovery and characterization. This approach predicted 1,675 new Gene Ontology (GO) terms across 783 genes, uncovering previously uncharacterized proteins linked to cellular aging. While these studies demonstrate the power of AI in accelerating genomic discovery, their predictions await experimental validation to confirm biological relevance.

#### 5.1.2 Aging assay automation incorporating microfluidics and AI

Traditionally, chronological lifespan (CLS) in yeast is measured by growing cells in liquid media until the post-diauxic phase, then plating aliquots every 1–2 days to count new colonies, while replicative lifespan (RLS) is determined by counting and separating each bud a mother cell produces until division ceases (Mirisola and Longo, 2022). A budding event forms a small bump, or bud, on the parent yeast cell, which grows and eventually detaches as an independent cell (Beran, 1968). These assays are labor-intensive, time-sensitive, and prone to human error in tasks like cell picking, daughter cell tracking, and image interpretation, leading to researcher-dependent variability (Thayer et al., 2022). To overcome these limitations, microfluidics and AI have been integrated into yeast aging studies. In 2021 (Ghafari et al., 2021) explored multi-model DL approaches, an ensemble of CNNs and Capsule Network (CapsNet), to classify yeast life stages from microscopic images, with 98.5% accuracy. Their study demonstrated that incorporating multiple architectures rather than single-model approaches, data augmentation and categorizing images into two broader life stages further improved classification performance. Notable advancements were made in 2022 with the integration of microfluidic technologies into this domain. Building on this, (Ghafari et al., 2022), introduced automated RLS prediction from microfluidic time-lapse images using ML approach. The system combined YOLOv3 detection, likelihood-based lineage tracking, and regression to predict RLS with 93% accuracy. While effective, this approach lacks the advanced spatial segmentation and scalability of Detecdiv, making it less adaptable for large-scale, high-throughput RLS analysis. Scalability is a crucial factor as high-throughput RLS analysis requires processing of large image datasets and limited scalability can comprise sample size and reduce statistical power (Thayer et al., 2022). introduced the ‘Yeast Lifespan Machine,’ a microfluidic platform employing ResNet-style and U-Net models to detect budding events and mother cell death respectively, revealing effects of genetics and pH on RLS. The system captures high-resolution images of mother cells, distinguishing them from daughter cells while monitoring genetic and environmental effects on aging (Aspert et al., 2022). engineered a microfluidic system named Detecdiv, which uses CNN for life phase classification, LSTMs for temporal tracking and DeepLabV3+ for spatial analysis of cell morphology, to automate RLS reconstruction and tracking single-cell division. Most recently, (Xiao et al., 2024), introduced the diploid yeast long-term culturing (DYLC) chip featuring 1,100 single-cell traps and an 18-layer ResNet algorithm, to extract RLS, budding time intervals, and dynamic events at high throughput (F1 > 92%) compared to the 24-channel ‘Yeast Lifespan Machine’ by Thayer et al. (2020). This system immobilizes mother cells while continuously removing daughter cells, enabling precise time-lapse imaging during replicative aging, marking a new benchmark in single cell aging research. Collectively, these microfluidic–AI platforms automate lifespan assays, minimize manual variability, and deliver high-throughput, precise insights into yeast aging.

#### 5.1.3 Key challenges

It is already evident how AI has not only enhanced biological assays in yeast and streamlined data management but has also provided deeper insights into the genetics of aging by identifying novel proteins and related genes. The most successful approach for yeast seemed to be integration of AI-based imaging and microfluidics, automating assays and enhancing throughput. However, a key limitation of these studies is the individual platform-based AI models (models trained for one platform might not work for other platforms) and lack of cross-species validation. While yeast serves as a powerful model for aging research, its ultimate goal is to inform human therapeutics. Experimental validation in higher organisms, both *in-vitro* and *in-vivo*, needs to be conducted across species in order to proceed towards human translation. Moreover, gene-discovery studies are conducted using genomic datasets which are restricted in sample size incurring a risk of data overfitting. AI-based microfluidic platforms often have unique architectures, requiring customized AI models tailored to each individual system. Differences in imaging and experimental protocols for the same biological parameter in such platforms generate datasets that are not compatible to train AI models across studies, limiting the generalizability of AI models. Establishing a standardized protocol for investigations of a given biological parameter can help address this issue and enhance reproducibility.

### 5.2 Caenorhabditis elegans

The transparent invertebrate nematode *C. elegans*, just 1 mm long, has revolutionized aging genetics. With a mere ∼20-day lifespan, cost-effective maintenance, and ∼65% of its genes having human orthologs, many of which are implicated in disease, this worm offers unparalleled insight into conserved longevity pathways (Lin et al., 2023; Zhang et al., 2020). Over years of extensive research aimed at promoting healthy and delayed aging, numerous genetic and signaling pathways with direct implications for aging have been discovered. Given its long-standing use in aging studies, comprehensive databases, including omics, chemical, and imaging resources, are available to support researchers in aging studies incorporating AI. The subsequent sections summarize pivotal AI-driven innovations in worm aging assays and lifespan analysis.

#### 5.2.1 Lifespan prediction

Age determination in *C. elegans* has traditionally relied on subjective visual assessment, but AI now enables accurate lifespan prediction and identification of aging-related mortality factors using image and video data. Due to aging being a multifactorial process, lifespan prediction can be approached from different perspectives. In this respect, (Martineau et al., 2020), employed multidimensional phenotyping and Support Vector Regression on high-resolution videos to predict age, remaining lifespan, and total longevity based on morphological and behavioral findings demonstrated accelerated aging in short-lived worms, with health deterioration proportional to aging rate. However, these conclusions are limited to wild-type worms, leaving open questions about genetic and environmental influences on aging trajectories. Mutant strains targeting specific genes reveal how individual genetic factors influence aging, providing broader insight into the pathways that regulate longevity. Therefore, incorporating diverse mutant strains and gene-silencing approaches is essential for comprehensive aging studies. To automate the physiological age determination, (Lin et al., 2021), integrated “curved_or_straight” attribute to InceptionResNetV2 model using day 1–14 worm images, achieving 87% accuracy, whereas (Safwan et al., 2023) modified the EfficientNet-B0 for video analysis, reaching 89.8% accuracy. Further innovations include (Song et al., 2022) Att-EfficientNet model trained on fluorescence microscopy images from the first day of adulthood till death, which in turn classified worms into six distinct lifespan stages with 72% accuracy. InceptionResNetV2 model is designed for powerful deep hierarchical feature extraction from complex images, whereas EfficientNet-B0 is a lightweight model used for scaling depth, width, and resolution of image in a balanced way to achieve strong accuracy with lower input resolution of image, and Att-EfficientNet further enhances EfficientNet model by focusing on the most informative parts of an image (Czaplewski et al., 2022). classified muscle aging stages (97.8% accuracy) based on self-learned features from IICBU image dataset using InceptionV3 model. Most recently, (Yakimovich and Galimov, 2021), introduced a two-stage DL framework combining U-Net-based HydraNet for segmentation and CNN-based WormNet for lifespan prediction, distinguishing short- (<7 days) and long-lived (≥8 days) worms. This was further aided by Class Activation Maps to localize key body regions influencing predictions. While these AI-driven approaches have advanced automated lifespan prediction, larger and more diverse datasets are needed to ensure broader reliability under varied imaging conditions (Kern et al., 2024). explored lifespan differences based on pathology progression by analyzing five key aging-related conditions: pharyngeal deterioration, intestinal atrophy, uterine tumors, pseudocoelomic lipoprotein pools, and gonad atrophy; along with variations in sex, nutrition, and genotype. Trained in lifespan and pathology data of *C. elegans* of days 1, 4, 7, 11, and 14, under different genetic conditions, RF achieved >70% accuracy in predicting. Their findings revealed sex-based lifespan differences and pinpointing pharyngeal and intestinal decline as strong mortality predictors, though generalizability remains limited by the small pathology set incorporated. A model trained on a small pathology dataset may not account for full spectrum of physiological deterioration leading to mortality across different genetic backgrounds or environmental conditions. As a result, the trained RF model may perform well within this specific dataset but is unlikely to generalize to broader *C. elegans* populations and other species without incorporating a more diverse and comprehensive range of pathologies.

#### 5.2.2 Uncovering molecular targets: predictive gene discovery

To identify lifespan-extending compounds, (Barardo et al., 2017; Ribeiro et al., 2023), developed predictive models using Random Forest (RF) models trained on DrugAge data, where the data catalogs chemical compounds with known effects on lifespan. Their models achieved ∼80% accuracy in prediction, identified nitroprusside as a promising anti-aging compound and highlighted that lifespan-extending compounds may target specific biological pathways, such as the glutathione metabolic process. However, external validation, conducted on independent DrugAge dataset, revealed possible overfitting and the absence of biological confirmation. Building on this computational pipeline for compound discovery, (Xie et al., 2022), applied the Mol2vec model to screen ChEMBL and ZINC databases for mitophagy inducers. The ChEMBL database contains biologically validated bioactive compounds while the ZINC database offers small molecules tailored for computational screening and drug discovery. Their model identified Kaempferol and Rhapontigenin, which were validated in *C. elegans* and mice, demonstrating neuroprotective and anti-aging potential. In a parallel study, (Truter et al., 2022), used a supervised ML pipeline (including Recursive Feature Elimination (RFE) with SVM and Generalized Linear Model) on multi-omics data to prioritize genes according to their contribution in aging. The findings claimed daf-2, let-363, rsks-1, and age-1 genes to be at the top of the priority list with some previously unknown potential targets for neurodegenerative diseases having orthologs in human. This study showcased ML’s ability to prioritize aging-related genes, but experimental validation and cross-species comparisons are needed.

#### 5.2.3 Aging assay automation

To accurately determine the assay termination, (García Garví et al., 2021), proposed an automated approach that combines CV with DL, specifically ResNet18 and LSTM, to classify worms as either alive or dead from low-resolution images with 91% accuracy. To mitigate the worm tracking issue, (Bates et al., 2022), developed a Faster R-CNN based model that accurately tracks worm movement across life stages and complex environments, although its generalizability is limited to certain strains and conditions. To improve data generalization while addressing data overfitting and labeling issues, researchers have increasingly adopted synthetic data. Beyond basic augmentation, generative adversarial networks (GANs) and reinforcement learning (RL) have been used for synthetic data generation (Moore et al., 2019). Notable examples include two distinct works by (García-Garví et al., 2023b; García-Garví et al., 2023a), who leveraged DL for synthetic image analysis using GANs to automate longevity prediction and lifespan assays. In the former lifespan prediction study, a bimodal neural network was trained on synthetic image data to predict the termination of lifespan curve hinting the end of assay. In the latter, they adopted a two-step approach combining Faster R-CNN for worm detection, and sequence-to-sequence neural networks (combining CNN with LSTM, GRU, and Transformer model) to count live nematodes over time. This pipeline reduced the manual effort of lifespan tracking as well as human error, achieving strong performance (F1 ≈ 0.9, MAE = 3.53%). Nevertheless, concerns persist about the applicability of synthetic data in real-world and genetic studies as such data may fail to capture true biological noise present in real images, rare phenotypes essential for reliable lifespan prediction and genetic interpretation along with introduction of potential artificial biases.

#### 5.2.4 Key challenges

The most prevalent approach generating reliable outcomes was models trained on vision and video dataset for prediction. While all these studies offer valuable insights into aging mechanisms, they often lack toxicity assessments. Furthermore, some studies specify the age of worms in microscopic images used for predictions, others do not. This lack of clarity affects reliability, as predicting aging in older worms is easier owing to their pronounced morphological and behavioral decline, whereas assessing early-stage aging is more challenging due to subtle phenotypic change and fast growth from one developmental stage to another. Thus, future improvements are necessary before these findings can be fully trusted and applied in real-world contexts.

### 5.3 Drosophila melanogaster

*Drosophila* has proven to be an ideal candidate for aging studies owing to its advantages including size (∼2–4 mm), cost-effectiveness, the ability to produce genetically identical offspring, a concise lifespan (∼40–120 days), and including orthologs for nearly 75% of human disease-related genes (Hirth, 2010). The key factor that sets *Drosophila* apart and maintains its relevance in aging research is its fair resemblance to human in terms of nervous system (Hirth, 2010; Tennant et al., 2024), reduced reproductive capacity (egg laying/fertility decline), declining motor and cognitive function, and cardiac dysfunction with aging (Lu et al., 2023). Additionally, advancements in genetic analysis tools have enabled comprehensive mapping of its genome, further enhancing its utility as a research model. The following sections review key AI applications in *Drosophila* aging studies, with an emphasis on translational insights and validation challenges.

#### 5.3.1 Longevity estimation

In a unique approach to lifespan estimation, (Zhang et al., 2021), estimated fruit fly lifespan by analyzing sleep patterns using a dual-step DL framework. First, zero-inflated autoregressive conditional Poisson (VZI-ACP) model was employed to calculate minute-by-minute activity probabilities for each fly, defining periods of inactivity as sleep states. Afterwards, these probabilities were converted into heat maps, serving as input for CNN model to classify flies into short and long-lived groups with >70% accuracy, surpassing conventional artificial neural network (ANN). This study not only linked age-related changes in sleep patterns to longevity but also suggested it as a biochemical aging marker. Shifting focus to age-related cardiac function decline, (Melkani et al., 2024), developed an automated DL pipeline combining UNet for cardiac video frame segmentation, CNN for direct age prediction from raw video frames, and a logistic regression model for analyzing cardiac parameters such as heart rate and systolic/diastolic diameters. Notably, the study predicted age with impressive accuracy, identified the dilated cardiomyopathy (DCM)-associated gene OGDH as a key regulator of cardiac function and disease detection.

#### 5.3.2 Precision aging clocks: cellular-level biological age estimation

For a comprehensive view of aging-related gene expression alteration, (Lu et al., 2023), developed a single-nucleus transcriptomic map named Aging Fly Cell Atlas (AFCA), followed by an aging clock for biological age estimation. The AFCA was constructed using snRNA-seq data from four age groups, revealing age-related shifts in gene expression, cell composition, and molecular pathways including downregulated cytoplasmic translation and ribosomal protein genes, upregulated lipid metabolism genes, metabolic remodeling in adipose cells, and selective alteration in neuronal signal transduction and protein phosphorylation pathways. To annotate cell types, the study employed supervised ML models, while regression models were used to develop an aging clock having single-nucleus transcriptomic as input. The clock identified ribosomal gene expression as a key driver of aging. A similar approach of developing aging clock for biological age-prediction using snRNA-seq data as input was attempted by (Tennant et al., 2024) but with a focus on sex-based differences in aging using DL. Unlike AFCA, which analyzed multiple cell types, this study concentrated exclusively on brain cells, employing a 1D Convolutional Neural Network (CNN) trained on snRNA-seq data. The model identified roX1, a long non-coding RNA (lncRNA) biomarker linked to X chromosome dosage compensation and sex-specific aging pathways. Notably, the model achieved a 94.3% accuracy in age prediction, and, unlike previous studies, validated its findings *in vivo* by conducting lifespan assay on roX1 loss-of-function mutant flies, which demonstrated reduced survival in the absence of this lncRNA.

#### 5.3.3 Mapping neurodegeneration: insights into brain aging

To investigate the biology of brain aging, researchers have turned to *Drosophila* due to its genetic and anatomical similarities to the human brain. To investigate age-related brain cellular changes, (Davie et al., 2018), constructed a single-cell transcriptome atlas of the adult *Drosophila* brain using Single-Cell RNA Sequencing (scRNA-seq), which profiled gene expression across the fly’s lifespan. Employing these gene expression profiles RF was trained for brain cell age prediction accurately. The study utilized SCENIC to identify aging-related gene regulatory networks, revealing an age-dependent RNA decline despite preserved neuronal identity. Lastly, they introduced SCope, an online platform for cross-species single-cell data analysis, that provided a rich, publicly available dataset for exploring brain aging and neurodegenerative processes in *Drosophila*. To identify conserved aging-related genes, (Webb et al., 2021), integrated RNA-seq data from both human prefrontal cortex (PFC) and *Drosophila* heads. Among 13 ML models tested, XGBoost performed best, revealing 50 age-associated genes linked to PI3K-Akt and MAPK signaling pathways, implicated in neurodegenerative diseases such as Alzheimer’s and Parkinson’s. Furthermore, the model classified flies and humans into three distinct age groups with notable accuracy.

#### 5.3.4 Insights into aging induced gene activity

Even though integration of AI in aging research gained significant momentum around 2019, for *Drosophila* it can be traced back to the early 2010s (Zhang et al., 2008). trained an SVM model, on time-series gene expression data across five distinct life stages of male fruit fly to predict Gene Ontology (GO) biological functions associated with specific genes. Using ten-fold cross-validation, the study examined gene activity across different body parts of aging fruit flies, such as the brain and gut, emphasizing the crucial role of mitochondria in the aging process.

#### 5.3.5 AI-driven insights into nutritional modulation of longevity

Beyond neurodegeneration, one of the most extensively studied factors influencing aging is caloric restriction (CR). While CR has long been recognized for its ability to slow aging, the underlying genetic mechanisms remain unclear. To address this gap, (Vega Magdaleno et al., 2022), trained four tree-based ensemble ML models (Balanced Random Forest, Easy Ensemble Classifier, XGBoost and CatBoost) on nine different biological features, including GO terms, biological pathways, and gene expression data, to determine whether aging-related genes are influenced by dietary restriction. Balanced Random Forest using GO term and CatBoost using biological pathways (PathDIP data) as the input, outperformed the others and successfully identifying seven genes as both age and diet related. While promising, the study lacks experimental validation. To further explore the relationship between caloric restriction, lifespan, and healthspan; (Hilsabeck et al., 2023); trained RF modeling on the *Drosophila* Genetic Reference Panel (DGRP) dataset, identifying key longevity-associated metabolites, including orotate, threonine, and choline. These findings were validated in humans using metabolomic data from the United Kingdom Biobank, suggesting that the Src64B gene plays a crucial role in regulating longevity factors under dietary restriction.

#### 5.3.6 Key challenges

The strongest AI contribution for *Drosophila* was omics data-based aging clocks, as they make reliable prediction of age as well as cell-type specific biomarkers, which can be experimentally tested. In addition to the common challenge of limited experimental validation, research using *Drosophila* as the model organism encounters comparatively more issues with limited and highly imbalanced datasets, as most studies were performed on small number of laboratory strains, specific tissues and age groups (Lu et al., 2023; Tennant et al., 2024). This leads to increased risk of data overfitting and biased results, reducing the reliability of findings. While these studies provide valuable frameworks for advancing aging research, further in-depth validation is essential before their conclusions can be fully trusted. Moreover, only a small subset of computationally identified biomarkers has been validated *in vivo* for (Tennant et al., 2024), but most studies have their predicted aging genes, markers and pathways unvalidated. Together, these drawbacks call for larger and more balanced datasets, standardized preprocessing and analysis protocols, and biological experimental validation of the AI-derived findings can be considered for human translation.

### 5.4 Mice

Mice serve as an exemplary mammalian model organism, characterized by a moderate lifespan (∼2 years), ease of manipulation, a well-defined connectome, and the availability of bioinformatic tools and genetic resources that facilitate the translation of findings to human aging research (Yuan et al., 2011). Mice exhibit a notably higher degree of similarity with humans compared to other model organisms in terms of genetics, physiology, immunology, nervous system, intricate brain and cardiac structures, making them particularly valuable for disease modeling like cardiac disorders and neurodegenerative diseases (Flurkey et al., 2007). The sections that follow highlight key AI-driven advances in murine aging research and outline future directions to strengthen translational impact.

#### 5.4.1 Longevity prediction

Early efforts on lifespan estimation date back to 2008, when (Swindell et al., 2008) trained a total of 22 different ML models on Mouse Phenome Database (MPD) and the ITP data, consisting of physiological markers including mice strain, sex, diet, body-type etc. Upon cross-validation, the Nearest Shrunken Centroid (NSC) algorithm demonstrated highest accuracy of only 35.3%, highlighting the need for improvements in data collection, sample size, and computational approaches. The limitations spurred further advancements in the field, leading to the development of more sophisticated age-detection algorithms, like (Brusini et al., 2022) developed a brain age prediction model from MRI images, identifying the gap between chronological and brain age as a potential aging biomarker. Using an ensemble of Gaussian Process Regression (GPR) and Logistic Regression, the model showed high accuracy, linking faster brain aging to higher mortality risk and slower brain aging to environmental enrichment and dietary restriction. However, cross-species validation is needed to confirm generalization.

#### 5.4.2 Multimodal aging clocks: methylation & lifespan forecasting

In 2020, (Levine et al., 2020), introduced DNAmAge, an epigenetic aging clock trained on Reduced Representation Bisulfite Sequencing (RRBS) data using Elastic Net regression, which predicted both chronological and biological age from DNA methylation patterns across tissues. Their findings linked increased methylation in heterochromatin regions to aging, with aged rats exhibiting increased locus-specific DNA methylation compared to younger counterparts, reinforcing the link between heterochromatin organization and epigenetic alterations. In the same year, (Schultz et al., 2020), developed two complementary aging clocks: FRIGHT (Frailty Inferred Geriatric Health Timeline) Clock for biological age prediction and AFRAID (Analysis of Frailty and Death) Clock for lifespan prediction, both based on Frailty Indices (health deficits such as symptoms and diseases) and trained with Random Forest regression. These models demonstrated that Frailty Indices can serve as reliable predictors of aging-related outcomes, offer scalable, non-invasive alternatives to methylation-based clocks for aging research. These frailty-based clocks demonstrated predictive accuracy comparable to DNA methylation clocks, with FRIGHT age predicting chronological age with a median error of ∼1.3 months and AFRAID estimating remaining lifespan with a median error of ∼1.7 months in mice, similar to the correlations reported for rodent DNA methylation clocks (r ≈ 0.9).

#### 5.4.3 Novel biomarker discovery for precision longevity

While age estimation remains a major focus of aging research, another critical area where ML has been applied is biomarker discovery. Contributing to this area, (Shi et al., 2021), aimed to identify novel aging-related metabolic markers through urine metabolomics analysis in rats. Using ultra-performance liquid chromatography/mass spectrometry for profiling urine metabolites and ensemble of LASSO regression and Support Vector Machine Recursive Feature Elimination (SVM-RFE) for biomarker identification, six key aging-related metabolites with potential as anti-aging therapeutics were revealed: epinephrine, glutarylcarnitine, L-kynurenine, taurine, 3-hydroxydodecanedioic acid, and N-acetylcitrulline. For example, taurine was later found to be regulating mitochondrial function, reduction in ROS, and inflammation; L-kynurenine demonstrated its implication in lifespan regulation. However, validating these metabolites in other species would strengthen their broader applicability.

#### 5.4.4 AI in neuroaging: metabolic & structural insights

Aging is one of the most significant contributors to neurodegeneration, prompting extensive research towards uncovering potential therapeutic strategies (Vallianatou et al., 2021). investigated aging-induced metabolic changes in specific brain regions and tested tacrine, a candidate drug for dementia treatment. Training an ensemble of Principal Component Analysis (PCA), Partial Least Squares Discriminant Analysis, Hierarchical Clustering Analysis, Multivariate Receiver Operating Characteristic analysis, and SVMs on mass spectrometry imaging (MSI) data, the effects of aging and tacrine on mitochondrial function, neurotransmission, and lipid signaling were accurately detected. Moreover, comparing metabolic alterations between young and middle-aged mice revealed an increase in neuroprotective antioxidants with age, alongside alterations in acetylcholine and L-carnitine pathways, suggesting region-specific metabolic shifts (MSI-based analyses revealed distinct age-related metabolic shifts across different brain regions) as potential biomarkers of brain aging. However, the study lacks biological validation, limiting the reliability of its findings. Approaching neurological deterioration from a different perspective, (McKenna et al., 2021), combined Multiple Particle Tracking (MPT) with a boosted decision tree model (XGBoost) to examine extracellular matrix (ECM) alterations due to aging and predict chronological brain age. MPT was used to track nanoparticle movement through the ECM, while XGBoost was trained on nanoparticle trajectory data to estimate brain age. The findings revealed that nanoparticle mobility declined with age, indicating a progressive increase in ECM density. This suggests that ECM alterations play a critical role in neurodevelopment and neurodegenerative disorders, but the study did not measure any neurodegenerative phenotypes by manipulating ECM, posing the proposed conclusion as a hypothesis requiring experimental validation.

#### 5.4.5 AI in deciphering age-driven molecular and structural alterations

Aging impacts tissues at different rates, as demonstrated by (Palmer et al., 2021), who trained an RF model on transcriptomic data (microarray and RNA-Seq) from humans, mice, and rats to examine age-related gene expression changes across tissues. Their meta-analysis identified that upregulation of immune and stress response genes drives brain aging, while downregulation of metabolic and developmental genes contributes to cardiac and muscle aging. Choi et al. (2022) developed an ML pipeline combining image processing with a Support Vector Machine Classifier (SVM-C) to investigate age-induced morphological changes in microglia. Trained on microscopic microglial images from three age groups of mice, the model categorized microglia into five distinct morphologies, revealing an age-related increase in activated microglia and a decline in rod-shaped types, indicating structural remodeling and potential involvement in neuroinflammation and neurodegeneration structural remodeling rather than proliferation while having a potential role in neuroinflammation and neurodegenerative diseases. In a related study on age-induced biochemical alterations, (Teker et al., 2023), investigated plasma exchange effects on liver tissues in young and old rats by infusing young plasma into old rats and *vice versa*. Using ATR-FTIR (Attenuated Total Reflectance-Fourier Transform Infrared) spectroscopy and analyzing the data with Linear Discriminant Analysis (LDA) and SVM models they found that young plasma infusion in old rats reduced hepatic fibrosis and cellular degeneration, whereas old plasma in young rats induced increased hepatic fibrosis, disrupted cellular organization, and steatosis. Extending this research to intestinal tissues, (Ceylani et al., 2023), used the same ML models and infrared spectroscopic data to assess ileum and colon responses. They observed rejuvenating effects of young plasma, particularly in ileum, including reduced protein oxidation, restored lipid-protein balance, and improved nucleic acid integrity. These findings support plasma-based interventions as promising regenerative therapy for reversing molecular aging across multiple tissues.

#### 5.4.6 Key challenges

Aging clocks and biomarker-identification models are the strongest AI contributions in case of mice, which are built from datasets like DNA methylation, frailty indices, imaging, and metabolomics, as mice are closely related to human physiology and disease processes than invertebrates. These models not only estimate biological age but also age-related health outcomes in a mammal. While mice offer many benefits over simpler model organisms due to their closer physiological relevance to humans, the common challenge faced by all these aging studies in mice was small sample sizes. Furthermore, the relatively long lifespan of mice (approximately 2 years) makes aging experiments time-consuming, which limits the rapid generation of large datasets. In addition, the high cost of animal maintenance and scaling restricts most studies to small cohorts, often resulting in sample sizes that are insufficient for robust AI model training and increase the risk of bias, noise sensitivity, and overfitting.

### 5.5 Human

Direct experimentation on humans to study aging is infeasible due to ethical considerations, complex human physiology and extended human lifespan. However, the integration of AI with extensive patient datasets offers a powerful alternative, enabling complex analyses and predictions from these large datasets without direct human testing. These insights can then be validated in other species and rigorously tested through multiple trials before potential translation to human applications.

#### 5.5.1 Age prediction from clinical and molecular data

Given the widespread use of various age-prediction models over traditionally used statistical methods, (Bae et al., 2021), compared conventional statistical models with four AI models: RF, XGBoost (XGB), Support Vector Regression (SVR), and Deep Neural Networks (DNN); using clinical biomarkers (e.g., blood pressure, metabolic markers, body composition metrics). Their findings confirmed DNN as the most accurate model for predicting biological age, showing the potential advantages of DL in complex aging predictions. Shifting the focus to investigating underlying factors of aging, (Wang et al., 2022), developed an ML-based biological age (BA) predictor using XGBoost algorithm on 44 clinical and physiological features (including blood biomarkers, lung function, and hearing threshold) to examine how lifestyle factors influence the aging process. The model showed strong correlation with chronological age (r = 0.82) and suggested that healthy lifestyle habits (such as maintaining a normal BMI, avoiding smoking and alcohol, exercising, and getting adequate sleep) may slow biological aging. Still, its reliance on self-reported data and the absence of molecular inputs limits its interpretability. Addressing aging from a molecular perspective, (Melendez et al., 2024), used elastic net algorithm for chronological age prediction via cerebrospinal fluid (CSF) proteomics data analysis, identifying underlying proteins and biological pathways associated with aging. While their model showed strong predictive performance and offered valuable insights into brain aging mechanisms, its clinical relevance remains limited due to small sample size and lack of biological validation.

#### 5.5.2 Progress in aging clock technologies

Contributing to the recent advancements in AI-powered aging clocks, (Sayed et al., 2021), introduced iAge, an inflammation-based immune aging predictor using a Guided Autoencoder (GAE) DL model. By analyzing blood immune biomarkers, iAge linked chronic inflammation to age-related conditions, including immune decline, frailty, and cardiovascular health, and identified CXCL9 as a key driver of cardiac and vascular aging. While the findings promote early detection of age-related disorders and personalized anti-aging interventions, clinical trials are needed to validate these claims. Extending aging clock development to ocular systems, (Ahadi et al., 2023), engineered eyeAge, a retinal aging clock using Inception-v3 architecture for chronological age prediction from fundus images. Achieving a mean absolute error of ∼3 years, eyeAge surpassed previous models and identified ALKAL2 as a top retinal aging gene via GWAS. Functional validation in *Drosophila* confirmed that ALKAL2 knockdown (via RNAi knockdown) delayed onset of age-related functional decline, supporting its role as a potential therapeutic target.

#### 5.5.3 Novel human aging biomarker identification

In an early effort to identify aging biomarkers, (Mamoshina et al., 2018), analyzed muscle gene expression data using five supervised ML models (ElasticNet regression, SVM, kNN, RF, and Deep Feature Selection) to estimate muscle tissue age. Their models identified 20 aging-related genes and pathways (e.g., PI3K-Akt-mTOR, PPAR signaling) and achieved a strong correlation (r = 0.91) between predicted and chronological age. Despite the novelty of their findings, limited sample size and lack of biological and independent datasets raise concern. Aging is often accompanied by increased blood glucose, decreased hemoglobin, alterations in cortical thickness and white matter structure, and a reduction in retinal cell counts (Gialluisi et al., 2019). Addressing this relationship between blood, brain, retinal biomarkers and aging, (Jonsson et al., 2019), developed a brain age prediction model using ResNet-based CNNs with transfer learning, analyzing T1-weighted MRI scans. They introduced the Predicted Age Difference (PAD), the gap between predicted structural brain age and chronological age, as a potential aging biomarker, where higher PAD reflects an older-appearing brain and has been associated with increased mortality risk and cognitive decline. They identified five brain-aging-linked genetic variants via genome-wide association study (GWAS). Yet, the study’s reliance on healthy subjects limits its clinical relevance for neural disorders. Expanding biomarker discovery beyond neuroimaging (Wang et al., 2020) identified circular RNAs (circRNAs) as forensic age biomarkers by applying multiple ML models to blood samples, with Random Forest Regression (RFR) achieving the highest accuracy and lowest MAE.

#### 5.5.4 AI-driven strategies for anti-aging gene and drug discovery

To classify proteins as aging-related or not, (Kerepesi et al., 2018), examined 21,000 human protein features using an ML pipeline incorporating XGBoost, SVM, and Logistic Regression. Their analysis validated established aging-associated proteins (e.g., BCL2, FOXO1, ERCC1, MTOR, SIRT2) while also proposing novel candidates like CY24A and ZC12A. Building on this, (Kulaga et al., 2021), combined linear regression models, SHapley Additive exPlanations (SHAP) and Bayesian networks to analyze the relationship between gene expression and maximum lifespan across five organs (brain, heart, kidney, liver, and lung) in 41 mammalian species. Their study uncovered 57 novel longevity-associated genes (e.g., DYRK4, NFKBIL1, TRAPPC2L, ETV2, CHCHD3) and key metabolic pathways while remaining purely computational and lacking biological validation. Addressing the strong association between aging and cancer, (Pun et al., 2023), utilized PandaOmics, an AI-driven multi-omics platform, to predict 23 previously unknown genes with both anti-aging and anti-cancer potential, alongside 22 experimentally validated targets. Notably, the KDM1A gene was tested in *C. elegans*, confirming its dual action in longevity and tumor suppression, warranting further translational research. Focusing on age-induced cardiac failure, (Yu et al., 2024), applied LASSO, RF, and SVM-RFE to gene expression data, identifying 14 key aging-related genes (10 upregulated, 4 downregulated) and predicted six potential drug candidates, with Rimonabant and Lovastatin emerging as the most promising. While imbalanced sample sizes and the absence of biological validation pose challenges, this study presents valuable insights into potential biomarkers for the early detection of cardiac failure.

#### 5.5.5 Key challenges

The most successful AI approach for humans is DL-based aging clocks trained on imaging data, because it worked well on large human dataset, generated biological age as output and could be easily compared across large cohort. Despite substantial progress, the application of AI and ML in human aging research faces critical limitations that must be addressed. While these tools have enabled the integration of clinical, molecular, and imaging data to uncover potential aging biomarkers and predictive models, most studies rely on datasets predominantly composed of healthy individuals. This introduces significant bias and limits the clinical applicability of findings to diverse or diseased populations. Furthermore, small sample sizes, lack of longitudinal data, and minimal representation of high-risk or elderly cohorts reduce the robustness and generalizability of these models. Additionally, many models lack integration of molecular-level data, which is essential for mechanistic interpretation and translational relevance. Together, these challenges underscore the urgent need for more inclusive datasets, biologically validated findings, and multimodal approaches to improve the reliability and clinical utility of AI in human aging research.

Both human and model-organism studies face limitations, but their drawbacks differ in nature. Like how human data for aging studies lack diversity, model organisms are constrained by small sample sizes. Moreover, human biology is influenced by numerous external factors (e.g., lifestyle, diet, and genetic variability), which introduce substantial noise into the data. In contrast, model organisms are maintained under tightly controlled experimental conditions, producing more consistent but less physiologically complex data that may limit direct translation to humans.

## 6 Discussion

AI has undoubtedly revolutionized aging research, transforming data analysis, predictability, pattern recognition, experimental acceleration, and application (Huang et al., 2023; Leung et al., 2024). The studies reviewed above clearly demonstrate how AI has not only contributed to novel discoveries and a deeper understanding of aging biology but has also significantly automated experimental procedures. A comparative overview of AI-based aging studies across the five model organisms-yeast, worms, flies, mice, and humans, featuring differences in AI techniques and dataset used, and advancements reported by the studies are reported in Table 2. Initially, AI in aging studies relied heavily on ML techniques, which faced challenges in terms of accuracy and validation. However, the integration of DL has significantly improved these outcomes (Aman et al., 2020; Bernal et al., 2024). Despite these advancements, it is important to remember that AI remains a tool rather than a standalone solution.

**TABLE 2:** Key AI methodologies and advances across aging model organisms.

Model system	AI technique	Dataset	Advancement	References
Yeast	Hierarchical clustering	Genomic	Unidentified longevity gene discovery	Chen et al. (2013)
	CNN and CapsNet ensemble	Microfluidic time-lapse images	Automation of yeast classification	Ghafari et al. (2021)
	2-layer predictive ML model with NN	Genomic	Effect of gene deletion, mitochondrial function and chromatin silencing on lifespan	Huang et al. (2012)
	ML predictor based on NET-FF	Colony-growth phenotypes	Uncovered uncharacterized proteins related to cellular aging	Rodríguez-López et al. (2023)
	CNN, LSTM, DeepLabV3+	Microscopy image	Developed microfluidic platform for automated yeast lifespan classification	Aspert et al. (2022)
	Division detection model (CV), 18-layer ResNet, YOLOv3, linear regression respectively	Microfluidic time-lapse images	Developed microfluidic platform for automated yeast RLS measurement	Ghafari et al. (2022), Thayer et al. (2022), Xiao et al. (2024)
*C. elegans*	SVR,InceptionResNetV2; EfficientNet-B0 model; InceptionV3; U-Net-based HydraNet & CNN-based WormNet respectively	Video, microscopic image	Lifespan prediction	Czaplewski et al. (2022), Lin et al. (2021), Martineau et al. (2020), Song et al. (2022), Yakimovich and Galimov (2021)
	RF	Image and video	Revealed pharyngeal and intestinal deterioration as key predictors of mortality	Kern et al. (2024)
	RF	DrugAge	Uncovered lifespan-extending compounds	Barardo et al. (2017), Ribeiro et al. (2023)
	Mol2vec	ChEMBL and ZINC	Discovered potent mitophagy inducers with anti-aging potential	Xie et al. (2022)
	Supervised ML pipeline	Multi-omics	Revealed novel targets for neurodegenerative disease	Truter et al. (2022)
	Att-EfficientNet, Faster R-CNN; Bimodal neural Network; ensemble of CNN + LSTM + GRU + transformer models, ResNet18 + LSTM respectively	Fluorescence microscopy image, Synthetic image	Lifespan assay automation-classification into lifespan stages; tracking movement; lifespan termination prediction; classification into dead or alive worm respectively	Bates et al. (2022), García Garví et al. (2021), García-Garví et al. (2023a), García-Garví et al. (2023b), Song et al. (2022)
*Drosophila melanogaster*	Regression and supervised ML models, 1D CNN	snRNA-seq data	Aging clock development- biological age estimation, sex- based differences in aging	Lu et al. (2023), Tennant et al. (2024)
	VZI-ACP + CNN	Numerical count of sleep pattern	Lifespan estimation	Zhang et al. (2021)
	UNet + CNN	Video	Automated analysis of cardiac dysfucntion and dynamics	Melkani et al. (2024)
	SCENIC; XGBoost	Gene expression profile; RNA-seq data respectively	Unveiled biology of brain aging- revealed an exponential RNA decline with age; discovered 50 conserved aging-related genes respectively	Davie et al. (2018), Webb et al. (2021)
	SVM	Gene expression profile	Discovered role of mitochondria in aging	Zhang et al. (2008)
	4 tree-based ensemble ML models- BRF + EEC + XGB + CAT; RF respectively	9 biological features + GO terms; DGRP respectively	Relationship between dietary restriction and aging- identified 7 genes as both age- and diet-related, identified key longevity-associated metabolites respectively	Hilsabeck et al. (2023), Vega Magdaleno et al. (2022)
Mice	NSC; XGBoost	Physiological and biochemical measurements; nanoparticle movement data	Lifespan prediction- physiological age; chronological brain age	McKenna et al. (2021), Swindell et al. (2008)
	GPR + Logistic Regression; PCA + Partial Least Squares Discriminant Analysis + Hierarchical Clustering Analysis + Multivariate Receiver Operating Characteristic analysis + SVM respectively	MRI images; Mass spectrometry imaging (MSI) data respectively	Brain study- brain-age prediction; aging-induced metabolic changes in specific brain regions respectively	Brusini et al. (2022), Vallianatou et al. (2021)
	Elastic net regression; RF respectively	RRBS data; Frailty Indices respectively	Aging clock- DNA methylation pattern analysis to explore the relationship between aging and epigenetic modifications; biological age + lifespan prediction respectively	Levine et al. (2020), Schultz et al. (2020)
	LASSO regression + SVM-RFE	Metabolomic Data	Biomarker discovery- metabolic	Shi et al. (2021)
	RF	Transcriptomic data	Examined age-related gene expression changes	Palmer et al. (2021)
	SVM-C	Microscopic image	Examined age-related morphological changes in microglia	Choi et al. (2022)
	ATR-FTIR + LDA + SVM	Infrared spectroscopic data	Blood plasma exchange on liver tissues; ileum and colon respectively	Ceylani et al. (2023), Teker et al. (2023)
Human	RF + XGB + DNN + SVR	Clinical biomarker	Comparative analysis between conventional statistical models with 4 AI models	Bae et al. (2021)
	XGBoost; Elastic net; ResNet-based CNN + transfer learning respectively	44 clinical and physiological features; cerebrospinal fluid (CSF) proteomics data; T1-weighted MRI scans respectively	Age prediction- biological age; chronological age; brain age respectively	Jonsson et al. (2019), Melendez et al. (2024), Wang et al. (2022)
	Guided Autoencoder; Inception-v3 respectively	Blood immune biomarkers; retinal fundus image respectively	Aging clock- predicted aging related chronic inflammation; chronological age prediction from retina respectively	Ahadi et al. (2023), Sayed et al. (2021)
	RFR; ElasticNet Regression + SVM + kNN + RF + Deep Feature Selection respectively	Blood Samples; Muscle gene expression data respectively	Circular RNAs as aging biomarkers; 20 aging-related genes and pathways for muscle aging respectively	Mamoshina et al. (2018), Wang et al. (2020)
	XGBoost + SVM + Logistic Regression	Human protein features	Classified proteins into aging-related and non-aging-related categories	Kerepesi et al. (2018)
	linear regression models + SHapley Additive exPlanations + Bayesian networks	Gene expression data	Identified 57 novel longevity-associated genes and key metabolic pathways influencing lifespan	Kulaga et al. (2021)
	PandaOmics	Omics data	Discovered genes with both anti-aging and anti-cancer potential	Pun et al. (2023)
	LASSO + RF + SVM-RFE	Gene expression data	Identification of aging-related genes linked to age-induced cardiac failure	Yu et al. (2024)

ML, machine learning; DL, deep learning; NN, neural network; NET-FF, feedforward neural network; CNN, convolutional neural network; LSTM, Long Short-Term Memory; CAPSNET, capsule network; CV, computer vision; YOLOV3, you only look once; Version 3; RF, random forest; SVR, support vector regression; ResNet, Residual Network; Faster R-CNN, Faster Region-based Convolutional Neural Network; ATT- Efficient NET, Attention-based EfficientNet; GRU, gated recurrent unit; SVM, support vector machine; SCENIC, Single-Cell Regulatory Network Inference and Clustering; XGBoost, Extreme Gradient Boosting; RNA-seq, RNA, sequencing; UNet, U-shaped Convolutional Neural Network; BRF, balanced random forest; DGRP, *drosophila* genetic reference panel; EEC, easy ensemble classifier; CAT, Categorical Boosting (CatBoost); NSC, nearest shrunken centroid; VZI-ACP, Zero-inflated autoregressive conditional Poisson; DNN, deep neural network; LASSO, least absolute shrinkage and selection operator; SVM-RFE, Support Vector Machine - Recursive Feature Elimination; ATR-FTIR, Attenuated Total Reflectance - Fourier Transform Infrared Spectroscopy; LDA, linear discriminant analysis; SVM-C, support vector machine with cost parameter; GPR, gaussian process regression; PCA, principal component analysis.

A key consideration in AI’s application to aging research is that its predictions and identifications are fundamentally dependent on the type and quantity of data used for training and validation (Aman et al., 2020; Bernal et al., 2024; Fabris et al., 2017; Gallistl et al., 2024). The dataset plays a crucial role in determining the efficiency and reliability of AI-generated results (Huang et al., 2023). When datasets are small, imbalanced, or lack critical or even subtle biological factors, the predictions made by AI models can be misleading (McDiarmid et al., 2018). For example, AI is widely used for identifying potential genes and proteins related to aging. However, if the dataset is not comprehensive or generalized, important genes or proteins may be overlooked (Theodorakis et al., 2024). To address this challenge, various AI models, including Generative Adversarial Networks (GANs) and reinforcement learning (RL), are employed to generate synthetic data (Leung et al., 2024). These models are designed to simulate real-world biological and imaging data in cases where there is data bias or limited availability (Bernal et al., 2024; Zhavoronkov et al., 2019). While synthetic data can compensate for data scarcity and closely mimic real-world data, it raises concerns about its realism, ethical implications, and data validation (Leung et al., 2024), posing potential risks of inaccuracies when applied to biological studies. This underscores the importance of wet-lab-based experimentation and biological validation in model organisms to complement AI-driven findings (Bernal et al., 2024; Fabris et al., 2017; Huang et al., 2023; Marino et al., 2023). A particularly striking finding is that only 3% of the studies included *in vivo* validation, emphasizing the need for implementation of a hierarchical model validation system: Level 1 (*in silico* only), Level 2 (*in vitro* confirmation), Level 3 (*in vivo* biological validation), as summarized in Table 3. Without completing this experimental validation hierarchy, AI-derived insights should be posed as hypothesis rather than translational (Aman et al., 2020; Marino et al., 2023).

**TABLE 3:** Unifying Framework integrating AI techniques with biological organization and hallmarks of aging.

Biological organization level	Aging hallmarks	AI applications	Common AI techniques	Example studies	References
Molecular (genes, methylation, proteins)	Genomic instability, Epigenetic alterations, Loss of proteostasis	Gene expression modeling, Methylation aging clocks, Proteomic-based prediction	Random Forest (RF), XGBoost, ElasticNet, SVM	DNAmAge clock in mice, CSF proteomics in humans, DrugAge modeling in worms	Levine et al. (2020), Melendez et al. (2024), Barardo et al. (2017), Ribeiro et al. (2023)
Cellular (cell state, behavior, morphology)	Mitochondrial dysfunction, Nutrient sensing deregulation, Cellular senescence	Cell image classification, Lifespan stage prediction, Single-cell transcriptomics	CNNs, U-Net, InceptionV3, WormNet, LSTM	HydraNet in *C. elegans*, CNN for muscle aging in worms, Microglia shape classification in mice	Yakimovich and Galimov (2021), Czaplewski et al. (2022), Choi et al. (2022)
Tissue/Organ (brain, heart, liver, gut)	Stem cell exhaustion, Intercellular communication changes	Cardiac function estimation, Brain age prediction, Tissue biomarker mapping	CNN (ResNet, Inception), UNet, Regression models	UNet + CNN for fly heart aging, MRI brain aging in rats, SVM-based metabolomics in liver	Melkani et al. (2024), Brusini et al. (2022), Shi et al. (2021)
Organismal/Systemic (whole body, lifespan)	All hallmarks combined	Biological age prediction, Lifespan and frailty modeling, Healthspan scoring	Deep Neural Networks (DNN), RF, Guided Autoencoders, Logistic Regression	iAge inflammation clock in humans, AFRAID & FRIGHT clocks in mice, Behavioral lifespan models in worms	Sayed et al. (2021), Schultz et al. (2020), Martineau et al. (2020)

**FIGURE 3:**
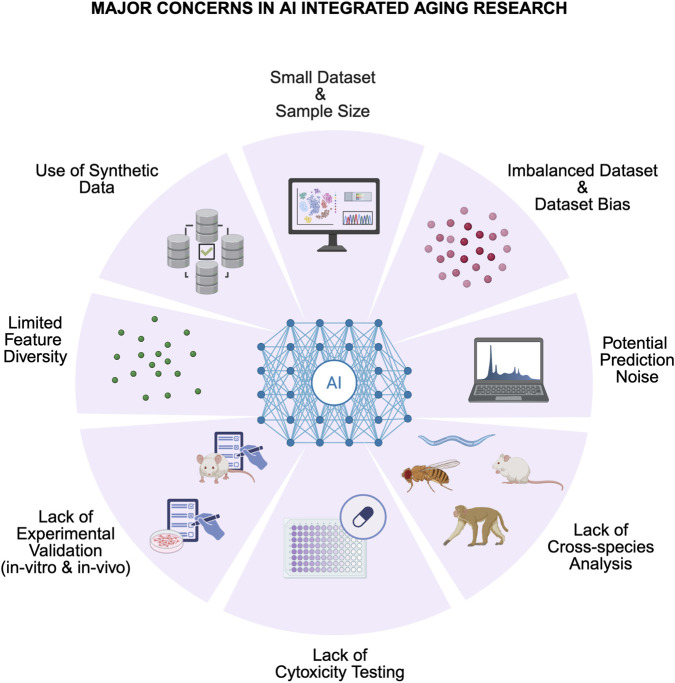
Major concerns in AI integrated aging research (created in https://BioRender.com).

Another challenge arises from the correlations AI models establish. Aging is a multifaceted process, influenced by numerous underlying factors. For example, if an AI model identifies an upregulation of a specific gene with aging, it does not necessarily imply that this gene causes aging. The gene may be affected by aging but not be a causative factor. As previously mentioned, the conclusions drawn by AI models are highly contingent on the data they are trained in. As a result, many drug candidates identified by AI models fail to pass clinical trials due to unforeseen side effects or lack of effectiveness. This reinforces the idea that while AI can provide valuable insights, it might not be causal and thus should not be fully relied upon for definitive conclusions (Fabris et al., 2017; Huang et al., 2023; Mishra and Li, 2020). An overview of these concerns is provided in Figure 3, illustrating the multifaceted limitations that currently restrict the broader clinical applicability of AI in aging studies.

Lastly, the utilization of longitudinal data in AI-based aging studies is currently underemphasized. Longitudinal datasets are obtained by repeated measurements from the same individual over time, which allows models to capture true aging trajectories. Using longitudinal data eliminates the potential inter-individual variability and produces more reliable aging predictions. In both human and model-organism studies, identification of early aging-linked changes, distinguishing cause from consequence, and validation of AI predictions by comparing predicted outcomes with actual future decline can be enabled by using longitudinal data. Thus, longitudinal data incorporation holds potential for strengthening model robustness, enhanced biological interpretability, and clinical relevance of AI-based aging research.

To conclude, the true power of AI in aging research lies in its ability to guide and enhance traditional scientific methods, rather than replace them entirely.

## 7 Future perspectives

As observed in the review, the field lacks a framework unifying AI technique to its corresponding application and biological mechanisms of aging. To bridge this gap, we propose an aligned framework, presented in Table 3, which clarifies how specific AI tool serves specific mechanistic ends better.

To enhance the trustworthiness and reliability of AI-generated findings in aging research, we propose the development of a standardized scoring system- AI Quality Assessment Metric (AI-QAM) for aging research. This scoring system would evaluate studies on six dimensions: (1) dataset size (number of data used to train a model), (2) feature dimensionality (techniques to maintain the relationship between number of input features and samples), (3) biological validation type (the three hierarchy levels mentioned in Table 4), (4) species diversity (how many species the AI findings tested on), (5) model generalizability (performance of models on different datasets, cohorts, and experimental setup), and (6) interpretability (linking AI model outcomes to biological concepts); providing a common assessment platform for AI contributions across the field. These metrics can be used by researchers to validate their results and submit the metrics score with their work, and reviewers can assess the claim and performance of the AI model based on a standard AI-QAM score. For example: each dimension can be scored from 0–3, making it a total of 18 across six dimensions. Moreover, this score can also be represented as percentage- %AI-QAM = (score/18) × 100. The scoring for individual dimensions can be done as mentioned in Table 5. For instance, the aging clock, eyeAge, developed by Ahadi et al. (2023) can be scored as: dataset size- 3, feature dimensionality- 3 (many pixels and feature extraction is well-defined in CNN), biological validation- 3 (*in vivo* in flies), species diversity- 2 (predicted using human data and validated in flies), model generalizability- 2 (tested on different cohorts) and interpretability- 2 (output linked to genetics via GWAS), so in total = 15/18. For FRIGHT + AFRAID aging clocks by Schultz et al. (2020), the scoring would be: dataset size- 2, feature dimensionality- 2 (manageable feature number, no high dimensional data), biological validation- 3 (*in vivo* in mice), species diversity- 1 (validation only in mice), model generalizability- 2 (tested on different mice dataset) and interpretability- 2 (frailty features are human understandable and important ones are mentioned by RF), so in total = 12/18. Lastly, the DNAmAge by Levine et al. (2020) can be scored as: dataset size- 2, feature dimensionality- 2, biological validation- 1 (correlated with biological outcomes), species diversity- 2 (developed in rats, tested in mice), model generalizability- 2 (tested on different mice dataset) and interpretability- 2, so in total = 12/18. The scores can be defined as, models generating a score of 9 or below can be considered as hypothesis-generating with moderate to low reliability; scores between 10–12 can be considered reasonably reliable and 13 and above is highly reliable with translational potential. The possible limitation of these metrics is that it is dependent on the reported information of the studies, which leaves possibility of scoring low despite good reliability if relevant information is not properly provided. Moreover, AI-QAM uniformly applies across all data types (omics, imaging, clinical). These data types vary in dataset size and feature complexity, which may lead to unfair strict scoring. A potential future refinement could be incorporating different data type-specific assigned scores.

**TABLE 4:** Experimental validation hierarchy.

Validation level	Type	Description	Example studies
Level 1	In silico	Only computer predictions or simulations	RF models trained on DrugAge for lifespan-extending compound prediction (e.g., nitroprusside prediction) (Barardo et al., 2017; Ribeiro et al., 2023)
Level 2	*In vitro*	Tested in lab models (e.g., cells)	None (like cytotoxicity testing)
Level 3	*In vivo*	Validated in living organisms	Validating roX1 gene in *drosophila* by (Tennant et al., 2024), ALKAL2 validation in C.elegans (Ahadi et al., 2023)

**TABLE 5:** AI-QAM scoring metrics.

Score	Dataset size	Feature dimensionality	Biological validation	Species diversity	Model Generalizability	Interpretability
0	Very small	Samples with no control	None	One species	No testing (only training performance mentioned)	None
1	Small	Samples with some control (feature filtering)	In silico	One species but in different strains and sexes	Tested on same dataset	Limited interpretation (lists features but does not connect them to biology)
2	Medium	Samples with explicit control (feature selection)	*In vitro*	Two species	Tested across different dataset	Clear interpretation (models link to genes, pathways)
3	Large	Samples with strong control (proper feature and overfitting management with explanation)	*In vivo*	Three or more species	Tested across different laboratories/experimental setup	Biology linked interpretation (experimentally proves the link of models to biology like gene knockdown or functional assays)

To summarize, this field requires not only advanced algorithms but smarter frameworks as well. Moreover, every AI study should pass through the experimental validation hierarchy mentioned previously to claim clinical relevance or translational potential. These measures will aid in transforming AI-driven insights into reliable, real-world interventions for aging.
